# Comparison of biological properties of umbilical cord-derived mesenchymal stem cells from early and late passages: Immunomodulatory ability is enhanced in aged cells

**DOI:** 10.3892/mmr.2014.2755

**Published:** 2014-10-23

**Authors:** YONG ZHUANG, DONG LI, JINQIU FU, QING SHI, YUANYUAN LU, XIULI JU

**Affiliations:** 1 Department of Pediatrics, Qilu Hospital, Shandong University, Jinan, Shandong 250012, P.R. China; 2 Cryomedicine Laboratory, Qilu Hospital, Shandong University, Jinan, Shandong 250012, P.R. China

**Keywords:** gene expression, immunomodulatory ability, senescence, umbilical cord-mesenchymal stem cell

## Abstract

Mesenchymal stem cells (MSCs) are a potential source of adult stem cells for cell-based therapeutics due to their substantial multilineage differentiation capacity and secretory functions. No information is presently available regarding the maintenance of immunosuppressive properties of this cell type with repeated passages. It was therefore the aim of the present study to analyze the biological properties, particularly the immunoregulatory effect, of MSCs from late passages. The differences between young and old MSCs in morphology, cell surface antigen phenotype, proliferation, gene expression and immunomodulatory ability were investigated. The results of the current study demonstrated that with the passage of cells, senescent MSCs displayed a characteristically enlarged and flattened morphology, different gene expression profiles and stronger immunosuppressive activities. Increased interleukin-6 production may be a possible underlying mechanism for this enhanced immunomodulatory ability of MSCs. These findings suggest that aged MSCs may provide a treatment option for patients with graft versus host disease and other diseases associated with dysregulation of the immune system.

## Introduction

Mesenchymal stem cells (MSCs) are a potential source of adult stem cells for cell-based therapeutics, due to their substantial multilineage differentiation capacity and secretory activity (1–4). Allogeneic umbilical cord and autologous bone marrow may be ideal practical sources of MSCs as they can be easily obtained without ethical concerns. Previous studies have provided encouraging results regarding the application of MSCs in tissue repair and regeneration in several disease models (5–8).

The immunomodulatory activity of MSCs is important in cell therapy (9); MSCs can directly modulate the function of T-cells. They inhibit the maturation and migration of various antigen-presenting cells, suppress B-cell activation, induce suppressor T-cell formation and alter the expression levels of several receptors required for antigen capture and processing (10,11). This immunosuppressive activity of MSCs is influential in tissue repair and regeneration. The anti-oxidative effects of MSCs can improve the survival of injured cells. In addition, expression of the heme-oxygenase-1 (HOX1) protein within MSCs has been demonstrated to decrease cytotoxicity and inhibit the apoptosis induced by oxidative stress (12).

Currently applied doses of MSCs range from 1 to 5 million MSCs/kg body weight (13). Thus, *ex vivo* expansion is required to meet the high demand of cell dose. With repeated passages, however, MSCs cultured *in vitro* inevitably undergo senescence, leading to reduced life span and growth arrest. While it has been demonstrated that aging may alter the capacity of MSCs to differentiate into osteoblasts or adipocytes (14), presently there is no information indicating the effects of aging on the immunosuppressive potential of MSCs.

Therefore, in the current study, MSCs from human umbilical cord (hUC-MSCs) were isolated, and their biological properties, particularly the immunomodulatory ability of hUC-MSCs, were compared between cells from early and late passages.

## Materials and methods

### Isolation and culture of MSCs

Human umbilical cords (n=10) were obtained from Qilu Hospital of Shandong University (Jinan, China) following clinically normal, healthy full-term deliveries. Informed consent was obtained from the parents of all individuals from whom tissues were collected. Tissue collection for research was approved by the Ethics Committee of Qilu Hospital (Shandong, China). Human umbilical cords were excised and washed in 0.1 M phosphate-buffered saline (PBS; pH 7.4) to remove excess blood. The cords were dissected, and blood vessels were removed. The remaining tissue was cut into small pieces (1–2 cm^2^) and placed in plates containing low-glucose Dulbecco’s modified Eagle’s medium supplemented with 10% fetal bovine serum, 100 U/ml penicillin and 100 μg/ml streptomycin (Gibco-BRL, Grand Island, NY, USA). Cultures were maintained at 37°C in a humidified atmosphere with 5% CO_2_. The medium was changed every 3–4 days. Following 7–12 days of culture, adherent cells were observed growing out from the individual tissue explants. The adherent fibroblast-like cells became confluent after 2–3 weeks of culture. They were treated with 0.25% trypsin (Gibco-BRL) and passaged at 1×10^4^ cells/cm^2^ in the medium described above. Cells at the third (hUC-MSC-p3) and fifteenth passage (hUC-MSC-p15) were analyzed in the following experiments.

### Cell morphology and scanning electron microscopy (SEM)

Cell morphology was observed daily under a light microscope (IX71 Olympus inverted microscope; Olympus, Tokyo, Japan) Wright-Giemsa staining was performed at the end of passage 3, 6, 9, 12 and 15. The nucleocytoplasmic ratio was analyzed by Image-Pro Plus software (version 5.1.0; Media Cybernetics, Rockville, MD, USA). hUC-MSC-p3 and hUC-MSC-p15 were fixed with 2.5% glutaraldehyde followed by post-fixing with 2% osmium tetroxide and 1% tannic acid. Following dehydration, cells were dried at critical point and lightly sputter-coated with platinum using an IB-50 ion-coater (Eiko Engineering Co., Ltd., Ibaraki, Japan). The samples were observed and photographed using a Hitachi S-570 Scanning Electron microscope (Hitachi, Tokyo, Japan).

### Cell surface antigen phenotype assessment by flow cytometry

hUC-MSC-p3 and hUC-MSC-p15 were collected and treated with 0.25% trypsin. The cells were individually stained with fluorescein isothiocyanate (FITC) or phycoerythrin (PE)-conjugated anti-marker monoclonal antibodies in 100 μl PBS for 15 min at room temperature, or for 30 min at 4°C, as recommended by the manufacturer. The antibodies used were specific for the following human antigens: CD34-PE, CD44-FITC, CD45-PE, CD73-PE, CD90-PE and CD105-PE (10 μl for 1×10^6^ cells; AbD Serotec, Raleigh, NC, USA). Cells were analyzed on a Cytometer 1.0, Cytomics™ FC500 flow cytometry system (Beckman Coulter, Brea, CA, USA). Positive cells were counted and the signals for the corresponding immunoglobulin isotypes were compared (15).

### Senescence-associated β-galactosidase (SA-β-gal) staining

SA-β-gal staining was performed using the Cellular Senescence Assay kit (Genmed, Shanghai, China). Briefly, cells were fixed for 5 min at room temperature in 1× fixing solution, washed and then incubated overnight at 37°C with fresh SA-β-gal staining solution. Cells were washed with PBS and then examined under a light microscope (IX71 Olympus inverted microscope; Olympus). A total of 10 visual fields were selected in the hUC-MSC-p3 and hUC-MSC-p15 samples, respectively, and the cell number per cm^3^ was calculated according to the average blue-stained cell number of the 10 fields.

### Cell proliferation assay of hUC-MSCs

hUC-MSCs were plated in triplicates at a density of 1×10^4^ cells/cm^2^ in 96-well culture plates. A 3-[4,5-dimethylthiazol-2-yl]-2,5-diphenyltetrazolium bromide (MTT) assay was performed daily for up to 6 days. Briefly, 20 μl of 5 mg/ml MTT (Sigma-Aldrich, St. Louis, MO, USA) was added to each well, and plates were incubated at 37°C for 4 h. The generated formazan was dissolved in 150 μl dimethyl sulfoxide and measured with a Model 450 microplate reader (Bio-Rad Laboratories, Richmond, CA, USA) at an optical density of 570 nm to determine cell viability.

### Microarray analysis and identification of differentially expressed genes (DEGs)

Microarray experiments were performed using Affymetrix GeneChip miRNA Array and Operating Software (GCOS; Affymetrix, Santa Clara, CA, USA) to analyze >41,000 known genes, gene candidates and splice variants. The two experimental groups were hUC-MSC-p3 and hUC-MSC-p15. Three independently cultured samples for each experimental group were used for the microarray analysis. GCOS was used for data collection and normalization. The log2-transformed signal ratio of each gene was calculated using the GCOS baseline tool to identify the DEGs. log2 ratio >2 (four-fold change) was used as the cut-off.

### Bioinformatic and functional analysis

For the functional analysis of DEGs, the gene ontology (GO; http://www.geneontology.org) functional categories and the Kyoto Encyclopedia of Genes and Genomes (KEGG; http://www.genome.jp/kegg/) functional pathways were searched for statistically enriched clusters/groups among the DEGs that were identified in the present study using the bioinformatics resources of the Database for Annotation, Visualization and Integrated Discovery (DAVID; http://david.abcc.ncifcrf.gov/). DAVID provides a comprehensive set of functional annotation tools for investigators to understand the biological meaning behind a large list of genes (16).

### Peripheral blood mononuclear cell (PBMC) proliferation assay

hUC-MSC-p3 and hUC-MSC-p15 were adapted to co-culture medium (RPMI-1640 medium supplemented with 10% fetal bovine serum, 2 mM L-glutamine, 100 U/ml penicillin and 100 μg/ml streptomycin) by gradually reducing the proportion of Dulbecco’s modified Eagle’s medium. Subsequently, MSCs were plated in triplicate in 96-well plates with 100 μl of co-culture medium at 100% confluence. Allogeneic PBMCs were isolated from peripheral blood by Ficoll-Hypaque gradient centrifugation (300 × g for 20 min), resuspended at 4×10^5^/ml, and then added to wells at a density of 4×10^4^ cells/well in 100 μl medium with or without MSCs in the presence of 10 μg/ml phytohemagglutinin (PHA, Sigma-Aldrich, Taiwan, China). After 48, 72 and 96 h, 100 μl of cells from each well were transferred to new 96-well plates containing 10 μl Cell Counting kit-8 reagent (Dojindo, Kumamoto, Japan). The absorbance at 450 nm was measured with a Model 450 microplate reader. All experiments were performed in triplicate and were repeated at least twice.

### Reverse transcription-quantitative polymerase chain reaction (RT-qPCR)

Total RNA was extracted from hUC-MSC-p3 and hUC-MSC-p15 using TRIzol reagent (Invitrogen Life Technologies, Carlsbad, CA, USA). To perform RT, first-strand complementary DNA (cDNA) was synthesized from 5 μg total RNA using Omniscript RT kit (Qiagen, Hamburg, Germany) according to the manufacturer’s instructions. PCR was performed using 2 μl tenfold diluted cDNA.

The cDNA samples were analyzed by RT-qPCR using the Applied Biosystems 7500 Real-Time PCR system (Foster City, CA, USA) with SYBR-Green I dye (Toyobo, Osaka, Japan). The primers (Table I) were obtained from Biosune (Shanghai, China). Data were analyzed using the 2^−ΔΔCT^ method, where ΔCT=(CT_target gene_-CT_β-actin_), to obtain the relative expression level, and each sample was normalized using β-actin expression. Results are presented as the fold change relative to the cDNA of hUC-MSC-p3. Data were analyzed using Sequence Detection system, version 1.4 (Applied Biosystems). Reported data are representative of at least three independent experiments.

### Interleukin (IL)-6 enzyme-linked immunoassay (ELISA)

The IL-6 levels in the supernatants of hUC-MSC-p3 and hUC-MSC-p15 were measured by ELISA (Mabtech, Nacka Strand, Sweden) according to the manufacturer’s instructions. IL-6 was assessed using three replicates of each sample.

### Statistical analysis

Data are presented as the mean ± standard error of the mean and assessed via analysis of variance and Student’s t-test. P<0.05 was considered to indicate a statistically significant difference All statistical analyses were conducted using the SPSS 13.0 software program (SPSS, Inc., Chicago, IL, USA).

## Results

### Biological characteristics of hUC-MSCs

**Figure 1:**
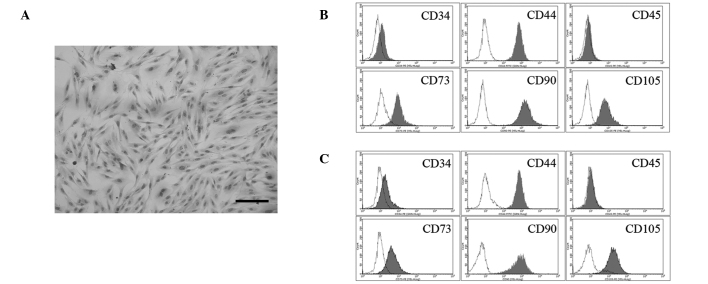
Morphology and immunophenotype of umbilical cord-derived cells. (A) Hematoxylin and eosin staining of umbilical cord-derived cells. Cells exhibited a bipolar spindle-shaped morphology and grew as a monolayer. Scale bar, 200 μm. Surface markers of (B) hUC-MSC-p3 and (C) hUC-MSC-p15 were detected by flow cytometric analysis. hUC-MSC-p3 and hUC-MSC-p15 expressed CD29, CD44, CD73, CD90, CD105 and CD106, but not CD34 and CD45, and no significant difference was identified between the expression levels (P>0.05). CD29 and CD106 are not shown in the figure.

Adherent cells with a fibroblastic morphology were observed from 48 h following establishment of explant cultures of human umbilical cord tissue (17). The cells formed a monolayer of homogeneous bipolar spindle-like cells with a whirlpool-like morphology within 2 weeks (Fig. 1A). The expression levels of cell surface molecules on the isolated cells were evaluated by flow cytometry for a minimum of three samples of hUC-MSC-p3 (Fig. 1B) and hUC-MSC-p15 (Fig. 1C). No significant differences in the percentage expression of any of the markers used for flow cytometric analysis were observed. The cells were positive for the following MSC markers: CD44 (99.3 and 99.2%); CD73 (77.8 and 74.2%); CD90 (94.9 and 98.1%); and CD105 (99.8 and 97.1% in hUC-MSC-p3 and hUC-MSC-p15, respectively). They were negative for the following hematopoietic markers: CD34 (0.86 and 0.41%); and CD45 (1.39 and 1.08% in hUC-MSC-p3 and hUC-MSC-p15, respectively), which is consistent with the phenotype of MSCs. hUC-MSC-p3 and hUC-MSC-p15 exhibited similar surface antigen phenotypes, and no significant difference was identified between them (P>0.05).

**Figure 2:**
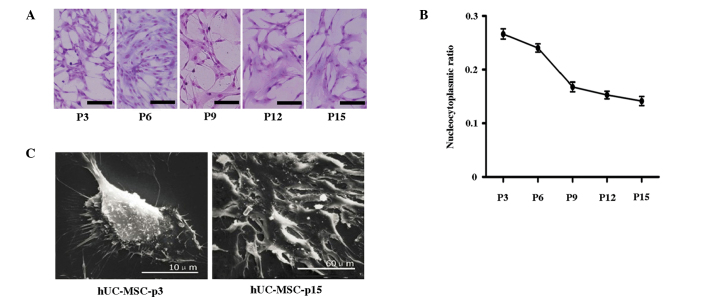
Morphologic changes in hUC-MSCs during long-term culture. (A and B) Giemsa staining of umbilical cord-derived cells. With the passage of cells, MSCs became flat, displayed (A) increased cell size, and (B) possessed reduced nucleocytoplasmic ratio. Scale bar, 100 μm. (C) Morphologic changes identified by SEM. hUC-MSC-p3 were plump and round in size and had equally distributed microvilli on the cell surface, whereas hUC-MSC-p15 exhibited more podia and less microvilli, spread more widely and contained more actin stress fibers. Scale bar: Left, 10 μm; right, 60 μm. MSC, mesenchymal stem cell; SEM, scanning electron microscopy; P, passage.

With the passage of cells, MSCs became flat with increased cell size, decreased nucleocytoplasmic ratio and increased presence of granulated cytoplasm and debris (Fig. 2A and B). As observed using SEM, hUC-MSC-p3 had a full shape and equally distributed microvilli on the cell surface, whereas hUC-MSC-p15 exhibited more podia and less microvilli, spread more widely and contained more actin stress fibers associated with cellular senescence (Fig. 2C).

### SA-β-gal activity and cell proliferation of hUC-MSCs

**Figure 3:**
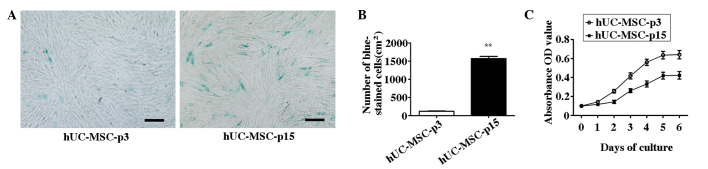
SA-β-gal activity and cell proliferation of hUC-MSCs. (A) Senescence of hUC-MSCs was detected by SA-β-gal staining. hUC-MSC-p15 exhibited high levels of SA-β-gal activity, as determined by the number of flattened and blue-stained cells. Few SA-β-gal-positive cells were observed in hUC-MSC-p3. Scale bar, 200 μm. (B) The number of blue-stained hUC-MSC-p15 cells were significantly higher than those of hUC-MSC-p3 (^**^P<0.01). (C) Cell proliferation was measured by MTT analysis. hUC-MSC-p15 exhibited a lower growth activity level than hUC-MSC-p3.

Analyses of senescence-associated SA-β-gal activity and cell growth were performed to demonstrate the senescence of hUC-MSCs. SA-β-gal activity is widely used as a biomarker for senescence. Results of the current analysis demonstrated that hUC-MSC-p15 exhibited a high level of SA-β-gal activity, as determined by the number of flattened, blue-stained cells, while few SA-β-gal-positive cells were observed in hUC-MSC-p3 (P<0.01) (Fig. 3A and B). With regards to cell proliferation, hUC-MSC-p15 had reduced growth activity compared with that of hUC-MSC-p3; thus, a greater number of hUC-MSC-p3 were present compared with the number of hUC-MSC-p15 (Fig. 3C).

### Identification of senescence-related genes in hUC-MSCs by microarray analysis

Whole-transcriptome oligonucleotide microarray analysis was performed to identify DEGs between early-passage (hUC-MSC-p3) and late-passage (hUC-MSC-p15) cells. Among the 44,000 probes represented on the microarray, 8,013 DEGs were observed in the comparison of hUC-MSC-p3 and hUC-MSC-p15 (data not shown).

Genes involved in deoxyribonucleotide catabolic process, small ribosomal subunit, snRNA binding, and NADH dehydrogenase activity were overrepresented in the upregulated genes of hUC-MSC-p15. By contrast, genes involved in the attachment of spindle microtubules to the chromosome, costamere, stress fiber and vinculin binding were overrepresented in the downregulated genes of hUC-MSC-p15. Subsets of representative significant GO terms of the up- and downregulated genes of hUC-MSC-p15 are presented in Table IIA and B, respectively.

The expression levels of gene sets containing curated pathways in the KEGG pathway database were compared with expression patterns in hUC-MSC-p3 and hUC-MSC-p15 using the Gene Set Enrichment Analysis algorithm to identify gene pathways with different expression patterns at different stages of aging. A significance threshold of P<0.05 to identify DEG pathways was applied. Using this threshold, 20 upregulated pathways were identified in the hUC-MSC-p15 samples. Among these pathways, seven were associated with metabolism and biosynthesis, and six were involved in autoimmune disorders and degenerative disease (Table III). Furthermore, 49 pathways were significantly upregulated in the hUC-MSC-p3 samples. The majority of them were associated with extracellular matrix (ECM), intercellular junction, metabolism, biosynthesis and cell proliferation (data not shown).

### Cellular senescence enhances the immunomodulatory activity of MSCs

**Figure 4:**
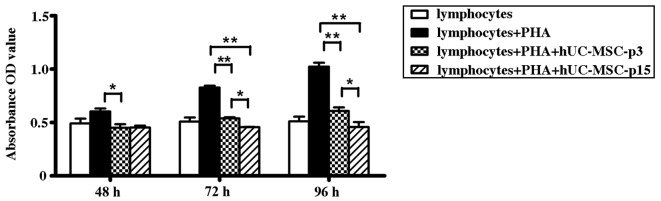
Senescence affects the capacity of cells to suppress allogeneic lymphocyte proliferation (^*^P<0.05 and ^**^P<0.01). hUC-MSC-p3 and hUC-MSC-p15 significantly inhibited PHA-stimulated peripheral blood mononuclear cell proliferation following 72-h co-culture (P<0.01), and the inhibitory activity of hUC-MSC-p15 was higher than that of hUC-MSC-p3 (P<0.05). Quantitative data are expressed as the mean ± standard error. PHA, phytohemagglutinin.

Immunosuppressive effects of MSCs from early and late passages were analyzed *in vitro* using a lymphocyte co-culture assay. MSCs (~1.9×10^4^) and PBMCs (~4×10^4^) were seeded into a 96-well plate. The results demonstrated that hUC-MSC-p3 and hUC-MSC-p15 were able to inhibit PHA-stimulated PBMC proliferation. Notably, hUC-MSC-p15 had a significantly stronger inhibitory effect on PBMC proliferation than hUC-MSC-p3 (P<0.05; Fig. 4).

### Changes in immunoregulatory-related cytokines during MSC senescence

**Figure 5:**
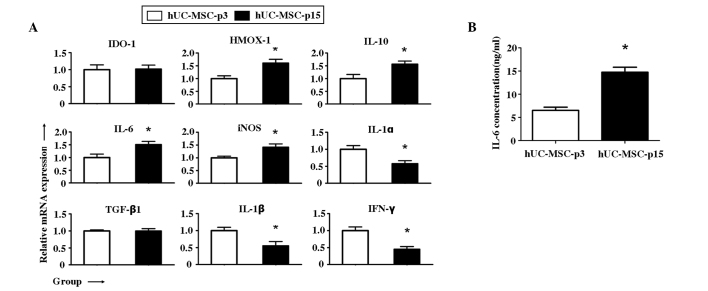
Changes in immunoregulatory-related cytokines. (A) RT-qPCR of immunoregulation-related genes (^*^P<0.05). The hUC-MSC-p3 group values were set at fold change=1. Four immunosuppressive genes, HMOX-1, IL-10, IL-6, and iNOS, were upregulated in hUC-MSC-p15 (P<0.05), whereas no significant difference was observed in IDO-1 and TGF-β1 (P>0.05). For the anti-inflammatory genes, IL-1α, IL-1β, and IFN-γ were downregulated (P<0.05). Quantitative data are expressed as the mean ± standard error. (B) ELISA results indicated that IL-6 production was greatly increased during senescence (P<0.05). HMOX-1, heme-oxygenase 1; IL, interleukin; iNOS, inducible nitric oxide synthase; IDO-1, indoleamine 2,3-dioxygenase-1; TGF-β1, transforming growth factor β1; IFN-γ, interferon γ.

In order to define whether cytokine production by MSCs was affected by senescence, the mRNA of nine immunoregulatory-related cytokines was measured by RT-qPCR. The following four immunosuppressive genes were upregulated in hUC-MSC-p15: HMOX-1; IL-10; IL-6; and inducible nitric oxide synthase (iNOS) (P<0.05). By contrast, indoleamine 2,3-dioxygenase-1 (IDO-1) and transforming growth factor-β1 (TGF-β1) were not identified to be significantly changed (P>0.05). The anti-inflammatory genes, IL-1α, IL-1β and interferon-γ (IFN-γ) were downregulated in hUC-MSC-p15 compared with levels in hUC-MSC-p3 (P<0.05) (Fig. 5A). Protein levels of IL-6 were also measured by ELISA, and the results indicated that it was significantly increased during senescence (P<0.05; Fig. 5B).

## Discussion

To consider the link between aging and MSCs, two interrelated components are important: *in vitro* aging (passage number in culture) and *in vivo* aging (donor age). Numerous studies have investigated the effects of donor age on MSC properties in addition to proliferation and differentiation capacities *in vitro* (18–20). Their findings indicate that *in vitro* aging has a greater effect on the proliferation and differentiation potential of MSCs compared with the effects of *in vivo* aging. In the current study, it was hypothesized that the basic effects of aging are associated with cellular senescence *in vivo* and *in vitro*. Therefore, in the present study, the effects of cellular senescence on the physiological, functional and molecular parameters of MSCs were focused upon. MSCs from the umbilical cord were cultured, and the fifteenth generation of cells were selected, mimicking cellular senescence.

Cellular senescence is a complex phenomenon that includes changes in functions and replicative capacities. Aging of cells during *in vitro* culture is dependent upon the number of cell divisions (21). With repeated passage, cells become enlarged and more granular, and their proliferation rates decrease. Ultimately, cells irrevocably stop dividing. Telomere shortening is currently established as one of the major mechanisms leading to replicative senescence (RS) (22,23). In addition, stress-induced premature senescence (SIPS) can be induced through several non-telomeric pathways involving cytokines, oncogenes (oncogene-induced senescence, OIS), persistent DNA damage activation or *in vitro* cell culture shock (24–26). In the present study, senescent MSCs were obtained through repeated passaging under normal atmospheric conditions. Therefore, RS and SIPS were assessed. hUC-MSC-p15 appeared as senescent cells with regards to morphology. Another sign of *in vitro* aging is a diminished division capacity due to reaching the maximal number of population doublings, which have been demonstrated to be 30–40 for MSCs *in vitro* (27,28). In the current study, hUC-MSCs exhibited high replicative potential until the fifteenth passage, when the cells gradually lost their proliferation potential. The number of SA-β-gal-positive cells, a marker for aged cells, increased in late-passage cells. However, with regards to the surface marker antigens, no significant age-related changes in the expression levels were observed, suggesting that the basic nature of the MSCs had not changed during senescence. Data from the current study demonstrated that MSCs at the fifteenth passage already had the characteristics of aging and therefore can be used as aged cells for further experiments.

Microarray analysis was used in the present study to compare the gene expression profiles of early-passage and late-passage hUC-MSCs, and to investigate the molecular mechanism underlying the senescence of these cells. Changes in cell morphology and function of senescent cells may partly be clarified by the microarray results. The gene expression levels of cytoskeleton-associated proteins, including actomyosin, stress fiber (Table IIB), fimbrin and microtubulin (data not shown) were significantly reduced. These proteins have functions in maintaining cell shape and maintaining internal structure; they are also involved in regulating a numebr of biological activities (29), including changing the stability and adaptability of the original external structure. The functions of transport vesicles and dynamic proteins inside the cells were suppressed. ECM was also downregulated in the aged cells. Given its diverse nature and composition, ECM can perform numerous functions, including providing support, segregating tissues from one another, and regulating intercellular communication and dynamic cell behavior (30,31). Their downregulation may be related to the metabolism, proliferation, migration and other aspects of functional changes in senescent cells. Increased metabolic levels of galactose, cholesterol synthesis and deoxyribonucleic acid, combined with decreased levels of protein synthesis and metabolism, may lead to the dysfunction and metabolic disorders observed in senescent cells. Several pathways associated with degenerative diseases were upregulated. The finding is consistent with individuals being more vulnerable to degenerative diseases with age.

It was also observed that a number of upregulated pathways were associated with autoimmune disorders. Considering that immunomodulatory activity is one of the key properties of MSCs, changes in the immunosuppressive potential of senescent MSCs were determined. The lymphocyte co-culture assay indicated that hUC-MSC-p15 had a significantly greater involvement in the inhibition of PHA-stimulated PBMC proliferation than hUC-MSC-p3. This result indicated that senescent MSCs may have a stronger immunomodulatory activity than young MSCs. MSC-mediated immunosuppression involves IDO (32), NO (33), IL-10 (34), TGF-β (35), and prostaglandin (PG) E2 (11). IDO-1 and cyclooxygenase-2 participate in the synthesis of PGE2 in MSCs. PGE2 is the major soluble factor responsible for the *in vitro* inhibition of allogeneic lymphocyte reaction (36). IDO-1 is an enzyme that catabolizes tryptophan, an essential amino acid, and is critical in MSC-mediated immunosuppression of various tissue origins (37). NO has a well-established role in macrophage function and has been demonstrated to affect T cell receptor signaling, cytokine receptor expression, and T cell phenotype (38). NO production is catalyzed by iNOS, which is important in immune regulation. IL-10, an anti-inflammatory cytokine, can directly regulate innate and adaptive Th1 and Th2 responses by limiting T cell activation and differentiation, and by suppressing pro-inflammatory responses in tissues, leading to impaired pathogen control and/or reduced immunopathology (39). HMOX-1 is an anti-inflammatory (40) and immunosuppressive molecule (41) in human MSCs (42) that can mediate the effect of molecules, such as IL-10 and NO (43). In the present study, HMOX-1 was indicated to be significantly upregulated in senescent cells. However, in contrast to a previous study by Toussaint *et al* (44), no significant changes were observed in the levels of TGF-β, a powerful pleiotropic immunosuppressive and anti-inflammatory cytokine (45). It is possible that the differences between the results are due to the use of different cell types or generations. In accordance with the changes above, three pro-inflammatory cytokines (IL-1α, IL-1β and IFN-γ) were significantly downregulated in senescent cells. These phenomena are in accordance with the results of lymphocyte proliferation assays. The upregulation of immunosuppressive and anti-oxidative molecules, together with the downregulation of proinflammatory cytokines, suggests that senescent MSCs have stronger immunosuppressive activities than less aged cells.

Specifically, it was noted that IL-6 mRNA was upregulated in senescent MSCs. IL-6 is theorized to function by triggering the suppressive effect of MSCs on T cells, as it induces the production of the anti-proliferative PGE2 in MSCs (46). In addition, neutralizing antibodies against IL-6 may reduce the capacity of MSCs to suppress T cell proliferation (46). These findings support the concept that IL-6 production is of relevance to immunoregulation by MSCs. Notably, IL-6 production has often previously been associated with aging (47,48), and IL-6 induces the expression of the GADD45a protein, which is associated with short telomere-induced replicative senescence (49). The results of the present study demonstrated increased IL-6 production by MSCs during senescence. This may explain why the suppression of PBMC proliferation by MSCs is enhanced following long-term *in vitro* culture.

In conclusion, changes in quantity, quality (differentiation/regeneration capacity) and immunomodulatory activity were observed between young and old hUC-MSCs. With the progressive passage of cells, senescent MSCs displayed a characteristic enlarged and flattened morphology, different gene expression profiles and stronger immunosuppressive activity. Increased IL-6 production may be a possible reason for the enhanced immunomodulatory ability of MSCs. The present study demonstrates further justification for the selection of MSCs in the treatment of immune-related diseases. However, the mechanism of cellular senescence and the effects of signaling pathways on immunomodulatory activities require further clarification.

## Figures and Tables

**Table I:** Primer sequences (5′-3′) used for reverse transcription-quantitative polymerase chain reaction.

Gene	Primer sequence
IDO1	F: GCCCTTCAAGTGTTTCACCAA R: GCCTTTCCAGCCAGACAAATAT
HMOX1	F: AGGGAAGCCCCCACTCAAC R: ACTGTCGCCACCAGAAAGCT
IL-6	F: GGTACATCCTCGACGGCATCT R: GTGCCTCTTTGCTGCTTTCAC
IL-10	F: GCCTTGTCTGAGATGATCCAGTT R: TCACATGCGCCTTGATGTCT
iNOS	F: GGTGGAAGCGGTAACAAAGG R: TGCTTGGTGGCGAAGATGA
TGFβ1	F: GGGAAATTGAGGGCTTTCG R: GAACCCGTTGATGTCCACTTG
IL-1α	F: GACGCCCTCAATCAAAGTATAATTC R: TCAAATTTCACTGCTTCATCCAGAT
IL-1β	F: GCGGCATCCAGCTACGAAT R: GTCCATGGCCACAACAACTG
IFN-γ	F: CCAACGCAAAGCAATACATGA R: TTTTCGCTTCCCTGTTTTAGCT
β-actin	F: GGACATCCGCAAAGACCTGTA R: GCATCCTGTCGGCAATGC

IDO1, indoleamine 2,3-dioxygenase-1; F, forward; R, reverse; HMOX1, heme-oxygenase 1; IL, interleukin; iNOS, inducible nitric oxide synthase; TGF, transforming growth factor; IFN, interferon.

**Table II:** Enriched GO terms in hUC-MSC-p15 samples.

A, Upregulated genes						

GO term	GO ID	Ontology	Observed genes (n)	Total genes (n)	Fold enrichment	P-value
Deoxyribonucleotide catabolic process	GO:0009264	BP	11	14	6.10	<0.001
Pyrimidine deoxyribonucleotide metabolic process	GO:0009219	BP	8	13	4.78	<0.001
Antigen processing and presentation of peptide antigen via MHC class I	GO:0002474	BP	11	18	4.74	<0.001
2′-Deoxyribonucleotide metabolic process	GO:0009394	BP	12	21	4.44	<0.001
Mitochondrial electron transport, NADH to ubiquinone	GO:0006120	BP	24	43	4.33	<0.001
Cytosolic small ribosomal subunit	GO:0022627	CC	22	35	4.90	<0.001
Organellar small ribosomal subunit	GO:0000314	CC	11	18	4.76	<0.001
Mitochondrial small ribosomal subunit	GO:0005763	CC	11	18	4.76	<0.001
Small ribosomal subunit	GO:0015935	CC	34	57	4.65	<0.001
Mitochondrial respiratory chain complex I	GO:0005747	CC	27	46	4.58	<0.001
snRNA binding	GO:0017069	MF	7	11	5.01	<0.001
NADH dehydrogenase activity	GO:0003954	MF	25	44	4.47	<0.001
NADH dehydrogenase (ubiquinone) activity	GO:0008137	MF	25	44	4.47	<0.001
NADH dehydrogenase (quinone) activity	GO:0050136	MF	25	44	4.47	<0.001
Oxidoreductase activity, acting on NADH or NADPH, quinone or similar compound as acceptor	GO:0016655	MF	26	50	4.09	<0.001

B, Downregulated genes						

GO term	GO ID	Ontology	Observed genes (n)	Total genes (n)	Fold enrichment	P-value

Attachment of spindle microtubules to chromosome	GO:0051313	BP	7	10	4.16	<0.001
NLS-bearing substrate import into nucleus	GO:0006607	BP	9	13	4.11	<0.001
Microtubule anchoring	GO:0034453	BP	15	22	4.05	<0.001
SMAD protein import into nucleus	GO:0007184	BP	9	14	3.82	<0.001
Mitotic chromosome condensation	GO:0007076	BP	8	13	3.66	<0.001
Costamere	GO:0043034	CC	8	12	4.15	<0.001
Condensed nuclear chromosome, centromeric region	GO:0000780	CC	7	11	3.96	<0.001
Actomyosin	GO:0042641	CC	19	34	3.48	<0.001
Stress fiber	GO:0001725	CC	16	29	3.43	<0.001
ER to Golgi transport vesicle	GO:0030134	CC	11	20	3.42	<0.001
Vinculin binding	GO:0017166	MF	7	10	4.18	<0.001
Mannosyl-oligosaccharide mannosidase activity	GO:0015924	MF	6	10	3.58	0.0025
DNA secondary structure binding	GO:0000217	MF	7	12	3.48	0.0013
Microtubule plus-end binding	GO:0051010	MF	6	11	3.25	0.0047
Poly-purine tract binding	GO:0070717	MF	6	11	3.25	0.0047
Mannosidase activity	GO:0015923	MF	8	15	3.18	0.0013

Subset of 15 representative significantly overrepresented GO terms of the upregulated and downregulated genes of hUC-MSC-p15. GO, gene ontology; CC, cellular component; MF, molecular function; BP, biological process; NLS, nuclear localization sequence; ER, endoplasmic reticulum.

**Table III:** Upregulated pathways in hUC-MSC-p15 samples.

Pathway ID	Definition	Enrichment score	P-value
hsa03010	Ribosome	18.62	<0.01
hsa05322	Systemic lupus erythematosus	16.50	<0.01
hsa00190	Oxidative phosphorylation	14.92	<0.01
hsa05012	Parkinson’s disease	11.46	<0.01
hsa05016	Huntington’s disease	8.94	<0.01
hsa05010	Alzheimer’s disease	8.32	<0.01
hsa05323	Rheumatoid arthritis	4.26	<0.01
hsa00100	Steroid biosynthesis	3.04	<0.01
hsa04621	NOD-like receptor signaling pathway	2.27	<0.01
hsa00052	Galactose metabolism	1.83	0.015
hsa03040	Spliceosome	1.82	0.015
hsa00900	Terpenoid backbone biosynthesis	1.75	0.018
hsa05132	Salmonella infection	1.74	0.018
hsa05164	Influenza A	1.66	0.022
hsa05133	Pertussis	1.59	0.026
hsa01040	Biosynthesis of unsaturated fatty acids	1.52	0.030
hsa04145	Phagosome	1.41	0.039
hsa04612	Antigen processing and presentation	1.38	0.041
hsa04330	Notch signaling pathway	1.36	0.044
hsa05219	Bladder cancer	1.35	0.045
